# Deep Vein Thrombosis in Lepromatous Leprosy: A Case Report of Secondary Pediatric Antiphospholipid Syndrome

**DOI:** 10.7759/cureus.21361

**Published:** 2022-01-18

**Authors:** Dyna Jones, Jayashankar CA, Amey Joshi, Shalini AS, Hemanth Kumar

**Affiliations:** 1 Internal Medicine, Vydehi Institute of Medical Sciences and Research Centre, Bangalore, IND; 2 Internal Medicine, Vydehi Institute of Medical Sciences and Research centre, Bangalore, IND; 3 General Medicine, Vydehi Institute of Medical Sciences and Research Centre, Bangalore, IND

**Keywords:** autoimmune, antiphospholipid syndrome, pediatric, leprosy, deep vein thrombosis

## Abstract

Antiphospholipid syndrome (APS) is a rare autoimmune disorder characterized by thromboembolic events, fetal loss during pregnancy, and evidence of antiphospholipid (aPL) antibodies such as beta-2-glycoprotein I (B2-GPI) and anticardiolipin (aCL). The diagnosis and treatment of this condition in the pediatric population have limited literature evidence due to the rarity of the condition in this age group. Guidelines have been adopted from the adult counterpart of the affected population, thereby giving rise to diagnostic and therapeutic challenges. In this report, we describe a rare case of a 15-year-old male who presented with lepromatous leprosy and developed deep vein thrombosis in his right leg. The laboratory evidence of positive aPL antibodies guided our diagnosis of APS and treatment with oral anticoagulants. This report highlights the importance of screening and timely diagnosis of APS in the pediatric population presenting with venous thrombosis in the backdrop of infection.

## Introduction

Antiphospholipid syndrome (APS) is a rare clinical entity characterized by an autoimmune process leading to thromboembolic events and recurrent fetal loss during pregnancy [1]. APS has been found to occur in about two persons per 100,000 per year, with an estimated prevalence of 50 per 100,000. The observed mortality in the affected population was noted to be similar to the general population [2]. The disease, although more commonly observed and extensively studied in the adult population, has limited evidence-based literature in the pediatric population. The assessment and diagnosis of pediatric APS are challenging due to the poorly validated diagnostic criteria and treatment guidelines based on literature from an adult-onset form of the disease [3]. Furthermore, the occurrence of APS secondary to a chronic granulomatous infection such as leprosy may further shroud the clinical presentation of venous thrombosis due to the peripheral neuropathic involvement and skin manifestation of leprosy. We present a case of a 15-year-old male who presented with lepromatous leprosy complicated with lower limb deep vein thrombosis that was later diagnosed as APS.

## Case presentation

**Figure 1:**
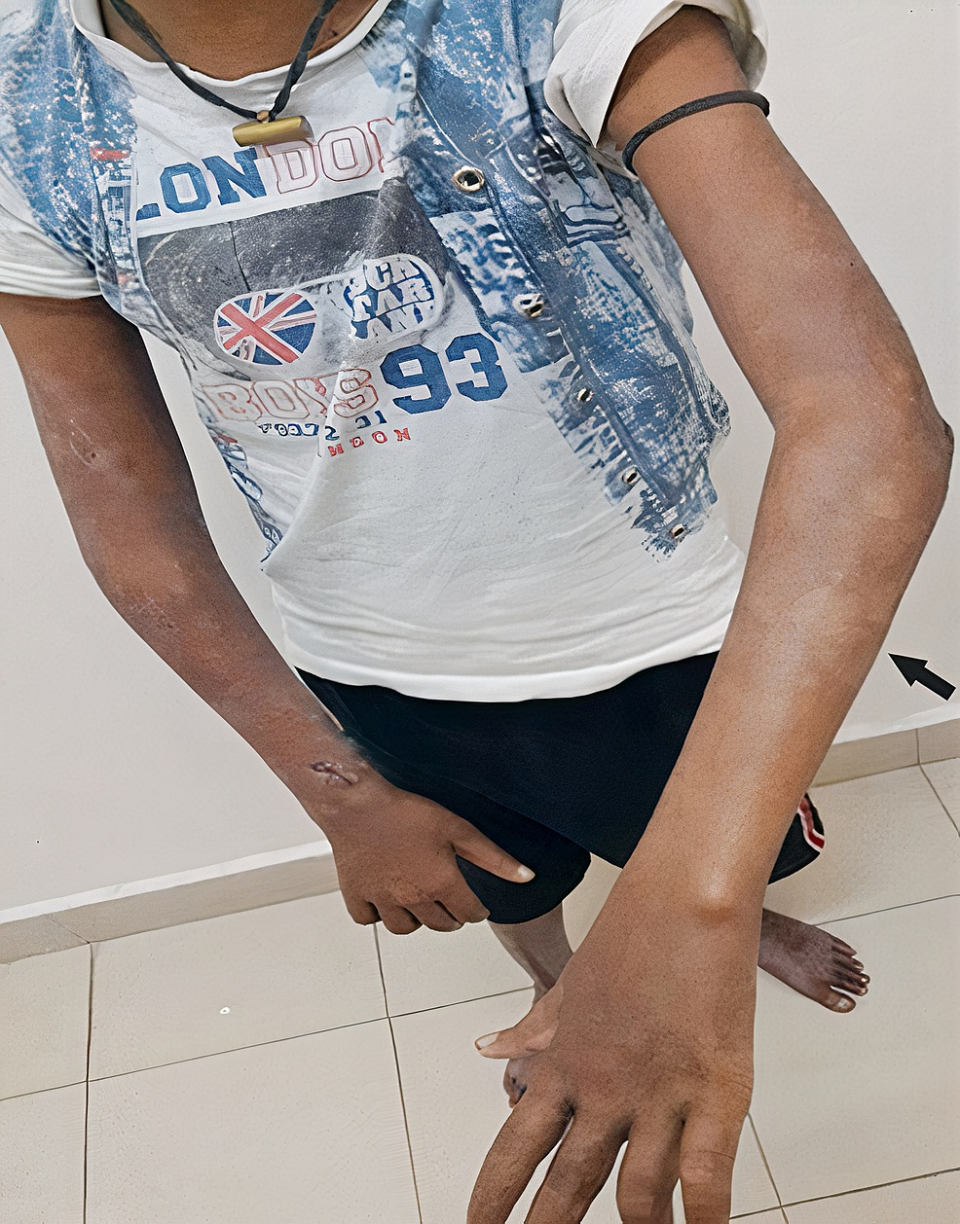
Multiple hypopigmented hypoesthetic macules noted on the extensor surfaces of upper limbs.

**Figure 2:**
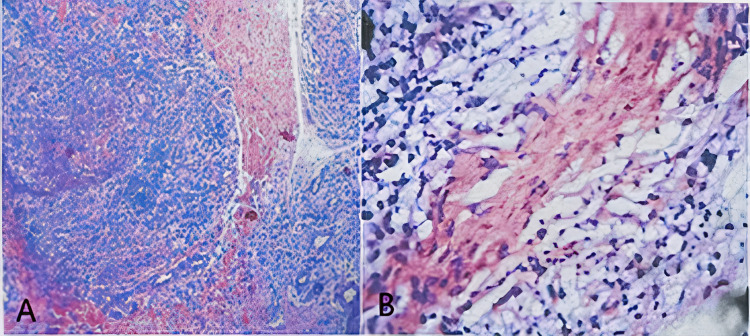
A: Hematoxylin and eosin preparation from skin lesion showing acanthosis and perivascular inflammation around small superficial and medium-sized vessels. B: Hematoxylin and eosin preparation at higher magnification showing lymphocytic predominant infiltration. (A and B, hematoxylin and eosin stain; original magnifications: A, ×10; B, ×40).

A 15-year-old male presented to our tertiary care hospital with a nine-month history of progressive bilateral upper limb and lower limb paresthesia. This was associated with weakness of the distal muscles of both the upper and lower limbs. On examination, the presence of multiple hypopigmented macules was noted distributed predominantly over the extensor surfaces of bilateral extremities, which the patient reported to have been persistent for five months (Figure 1). Clawing of the fourth and fifth digits of bilateral hands, bilateral atrophy of hypothenar muscles, and the absence of bilateral ankle reflexes were noted. In addition, we observed that the patient had a bilateral sensory loss to touch and temperature in a glove and stocking distribution up to the mid-forearm and midleg. Nerve conduction study (NCS) was suggestive of asymmetric severe sensory-motor axonal peripheral neuropathy involving all four limbs, with features consistent with mononeuritis multiplex. Punch biopsy of the skin lesions showed keratotic skin, acanthosis, and aggregation of foamy cells around vasculature and nerve twigs. Perivascular inflammation around superficial and medium-sized vessels was noted, comprising predominantly of lymphocytes (Figure 2). Modified Fite Faraco stain showed numerous lepra bacilli in singles and globi within the foamy cells and tissue consistent with lepromatous leprosy. Ultrasound of the abdomen was suggestive of mild right-sided hydronephrosis; however, further workup of this revealed no obstruction and normal urine microscopic evaluation. The chest X-ray and 2D echocardiography were unremarkable.

We commenced our patient on a regimen of rifampicin, dapsone, and clofazimine. During the course of the treatment, our patient developed dull-aching pain in his right leg. An arterial and venous Doppler was done, which revealed a thrombosed intramuscular vein in the anterior compartment of the right leg with minimal subcutaneous edema in the right ankle and foot. The bilateral lower limb arterial system appeared normal. Coagulation profiles revealed an elevated activated partial thromboplastin time (aPTT) of 42.1 seconds (normal range: 30-40 seconds). The international normalized ratio (INR) was 1.13. Laboratory evaluation demonstrated high lupus anticoagulant titers and beta-2-glycoprotein I (B2-GPI) IgM titers. Complement levels (C3 and C4), ANA titers, RA factor, antistreptolysin O (ASO) titers, and anticardiolipin (aCL) antibodies were negative. Liver enzyme tests were unremarkable, with an aspartate aminotransferase (AST) and alanine aminotransferase (ALT) of 56 IU/L and 50 IU/L, respectively. Meeting the revised Sapporo criteria, we diagnosed our patient with APS.

The patient was started on heparin with a pediatric-adjusted dose for bridging and transitioned to oral anticoagulation with acenocoumarol that was titrated to maintain an INR of 3. We observed a spontaneous resolution of the deep vein thrombosis over one week. The patient was continued on oral anticoagulation for three months duration with close follow-up. The patient was symptomatically better with the antileprotic medications and anticoagulation. No further episodes of venous thrombosis were observed during the follow-up period.

## Discussion

APS is defined as a systemic autoimmune disorder characterized by arterial and/or venous thrombotic events with positive antiphospholipid (aPL) antibodies. This disorder has been extensively studied in the adult population, and guidelines for diagnosis and treatment have been well defined [1]. APS, in the pediatric population, however, has limited literature evidence due to the rarity of the condition. Thus, the challenges begin, and early diagnosis of pediatric APS that often does not fit into the guidelines becomes challenging. Beyond the diagnosis of pediatric APS, the treatment guidelines lack definitive evidence, and the choice of anticoagulation has not yet been clearly defined. The study case describes a young male diagnosed with leprosy, which was complicated by the development of new-onset APS that was managed with anti-leprotic medications and timely anticoagulation.

Pediatric APS is defined as the syndrome presenting before the age of 18 years. To diagnose APS, the presence of at least one proven clinical event (arterial thrombosis/venous thrombosis/pregnancy morbidity) and laboratory evidence of at least one persistently positive aPL test (>12 weeks positivity of lupus anticoagulant (LA), anticardiolipin (aCL) or anti-beta-2-glycoprotein I (anti-B2-GPI) IgG or IgM) are necessary. Contrasting to the female preponderance in the adult population, pediatric APS has been found to occur almost equally in males and females [3]. The Pediatric APS (Ped-APS) Registry has been the largest cohort comprising 121 patients. The mean age of onset of APS in these patients was found to be 10.7 years. Secondary APS was noted to occur in children with coexisting conditions such as SLE, juvenile idiopathic arthritis (JIA), juvenile dermatomyositis, Henoch-Schönlein purpura, autoimmune thyroiditis, hemolytic-uremic syndrome, Behçet's disease, rheumatic fever, and malignancy [4]. In the same registry, it was also noted that of the 88 children with an upper airway infection, 30% of them had positive anticardiolipin antibodies. There was an even greater association of infection being the precipitator of catastrophic APS in children as compared to adults (60.9% versus 26.8%). Leprosy, however, was not noted to be a precipitator in any of these cases [5].

Multiple hypotheses have been made trying to understand the pathophysiology of thrombosis in APS. The binding of membrane phospholipids of cells with defective apoptotic mechanisms to circulating plasma proteins, such as beta-2-glycoprotein 1, have been found to result in autoantibody production to the formed neoepitopes. Other mechanisms include the production of antibodies to coagulation factors such as prothrombin, protein C, protein S, and annexins, activation of platelets to endothelial cells, and complement activation [5,6]. An underlying genetic basis of APS has also been defined, and HLA DR and HLA DQ have shown a consistent association with the syndrome. Antiphospholipid antibodies are frequently associated with other autoimmune diseases. The presence of aPL antibodies in infection was noted as early as 1999 in a patient with syphilis. Later, aPL antibodies were observed in other bacterial and viral infections such as HIV, hepatitis C, and leprosy. In a cohort of 51 patients with leprosy, 68.6% (n = 35), 60.7% (n = 31), and 56.8% (n = 29) had lupus anticoagulant (LA) antibodies, anticardiolipin antibodies, and anti-B2-GPI, respectively [7].

The risk of thrombosis in APS is the highest in triple positivity (LA positive, aCL positive, and anti-B2-GPI-1 positive) [8]. Observational studies of aPL-positive children with lupus have demonstrated an annual thrombosis incidence ranging from 3.1% to 6.6% [9]. The incidence of venous and arterial thrombosis was identified first in the Ped-APS Registry [4]. Of the children, 60% were found to develop venous thrombosis, of which deep vein thrombosis in the lower limb was the most common single event occurring in 40% of the patients. Other manifestations included venous thrombosis in the cerebral venous sinus (7%), portal vein (3%), upper extremity (2%), superficial veins (2%), and left atrial thrombus (2%). Arterial thrombosis was observed at a lower proportion of 32%, in which ischemic stroke was the most common manifestation occurring in 79% of these individuals [4]. In this study, our patient developed thrombosis of the intramuscular vein in the anterior compartment of the right leg. The lack of preexisting risk factors in our patient, such as a prior history of thrombosis, hypertension, and smoking, and the negative workup for protein C and S deficiencies and other thrombophilia helped us attribute the cause of the thrombosis to the underlying APS.

The association between leprosy and APS received attention in the late 20th century. It was found that patients with leprosy showed a wide spectrum of antibodies in their sera, such as rheumatoid factor, anti-DNA, antinuclear, and anti-mitochondrial antibodies, due to the polyclonal activation of antibody response. Some authors suggested that autoantibodies in leprosy originated not because of an autoimmune phenomenon but because of the molecular mimicry due to the cross-reactivity between *Mycobacterium leprae* antigen and self-antigens. Interesting to note was the close association researchers found between anti-B2-GPI IgG and venous thrombosis, which was largely greater than that observed with patients with positive IgM titers of the same antibody [7]. In the present study, we observed venous thrombosis in the presence of the latter.

Early initiation of treatment is vital to reduce the morbidity associated with venous thrombosis in pediatric APS. Due to the limited available data regarding pediatric APS and its management, treatment guidelines have been adapted from literature evidence of adult-onset disease. The Single Hub and Access Point for Pediatric Rheumatology in Europe (SHARE) initiative in 2017 published treatment guidelines for any child with positive aPL antibodies and evidence of venous thrombosis. Vitamin K antagonist such as warfarin was described as the primary modality of treatment of thrombosis in this population. INR was to be targeted to 3.0-4.0, and in the case of persisting aPL antibodies, long-term anticoagulation was recommended. In the case of arterial thrombosis, it was also recommended to include antiplatelet agents such as aspirin and clopidogrel alongside long-term anticoagulation [10]. Data is limited with the use of low-molecular-weight heparins, unfractionated heparin, and newer oral anticoagulants. Another disadvantage of using heparin is the parenteral mode of drug administration, which children may not be compliant with [10]. Our patient was administered heparin initially and warfarin, which was slowly titrated to achieve the target INR of 3. During the hospital stay, he was administered anti-leprotic medications to treat the underlying leprosy and concurrently managed with anticoagulation for the venous thrombosis.

## Conclusions

This case highlights the rare occurrence of pediatric APS secondary to leprosy. Leprosy, an endemic disease in certain parts of the world, must involve careful evaluation and consideration of the potential venous thrombosis secondary to APS. Timely interventions through radiological diagnostics such as Doppler scans and thrombophilia workup are warranted in such patients. Increased aPTT during the workup of these patients necessitates further workup of potential APS. Vitamin K antagonists remain the mainstay of deep vein thrombosis in secondary pediatric APS, and larger randomized control trials are required to ascertain the use of NOACs and heparin in such patients.
